# Reduced Emergency Department Visits and Hospitalizations in Infants after Universal Respiratory Syncytial Virus Immunization, Italy, 2024–25

**DOI:** 10.3201/eid3201.250870

**Published:** 2026-01

**Authors:** Simone Villa, Simona Scarioni, Enrico Pigozzi, Manuel Maffeo, Mauro Maistrello, Giorgio Bagarella, Francesco Scovenna, Federica Morani, Marlen Romano, Gianvincenzo Zuccotti, Massimo Agosti, Catia Borriello, Elena Pariani, Guido Bertolaso, Luigi Vezzosi, Fausto Baldanti, Mario Melazzini, Elena Azzolini, Gabriele del Castillo, Danilo Cereda, Regional Immunization Council

**Affiliations:** Directorate General for Health, Lombardy Region, Milan, Italy (S. Villa, S. Scarioni, E. Pigozzi, M. Maffeo, M. Maistrello, G. Bagarella, F. Scovenna, F. Morani, C. Borriello, G. Bertolaso, L. Vezzosi, M. Melazzini, G. del Castillo, D. Cereda); University of Milan, Milan (S. Villa, S. Scarioni, E. Pigozzi, F. Scovenna, G. Zuccotti, E. Pariani); Regional Company for Innovation and Purchasing Aria S.p.A., Milan (M. Romano); Buzzi Children’s Hospital, Milan (G. Zuccotti); Hospital “F. Del Ponte,” University of Insubria, Varese, Italy (M. Agosti); Fondazione IRCCS Policlinico San Matteo, Pavia, Italy (F. Baldanti); University of Pavia, Pavia (F. Baldanti); Humanitas University, Pieve Emanuele, Italy (E. Azzolini)

**Keywords:** respiratory syncytial virus, viruses, respiratory infections, respiratory viruses, surveillance, syndromic surveillance, immunization, Italy

## Abstract

During the 2024–25 winter season, a universal immunization campaign with nirsevimab was implemented in a region of Italy to prevent respiratory syncytial virus (RSV) infection among infants <12 months of age. We assessed its effects using regional syndromic surveillance data on emergency department visits (EDVs) and hospitalizations for lower respiratory tract infections and RSV infections. We estimated expected burden using an interrupted time series analysis, based on historical trends, and observed values with predictions. Children 1–5 years of age, not eligible for immunization, served as a comparison group. Among infants, EDVs for acute lower respiratory tract infections decreased by 42.7% and hospitalizations decreased by 46.5%, whereas EDVs for RSV infection decreased by 49.3% and hospitalizations decreased by 55.0%. No reductions were observed in children 1–5 years of age, confirming ongoing RSV circulation. Our findings support the effectiveness of universal nirsevimab immunization in reducing severe RSV-related outcomes among infants.

Respiratory syncytial virus (RSV) is the most common cause of acute lower respiratory tract infections (LRTI) in children and can potentially result in bronchiolitis, which often requires hospitalization. Young children, especially during their first 6 months of life, are at high risk for illness and death caused by RSV, particularly during a first infection (*1*).

Nirsevimab (Beyfortus; AstraZeneca, https://www.astrazeneca.com; Sanofi Pasteur, https://www.sanofi.com), a long-acting monoclonal antibody (mAb) against RSV, has an extended half-life of ≈71 days (*2*) and has been used in recent years to prevent severe RSV infections and outcomes in children <1 year of age. Recent clinical trials have demonstrated its efficacy in preventing RSV-associated acute LRTI, with an observed efficacy of 75% (*3*). In addition, a 62% reduction in hospitalization because of RSV-associated LRTI has been reported (*3*), which increased to 78.4% among preterm infants (*4*). A pragmatic trial conducted under near real-world conditions showed 83.2% overall effectiveness of nirsevimab and 75.7% effectiveness in reducing severe RSV-associated LRTI requiring supplemental oxygen (*5*). Other studies using real-world data have reported similar results, especially for RSV-related hospitalizations and severe RSV disease (*6*–*8*). A systematic review and meta-analysis has further validated that mAb administration in infants reduces the burden of RSV-related hospital and ICU admissions (*9*).

Initial experiences with routine nirsevimab immunization in infants have been reported in several hospitals across Europe (*10*–*12*), and new population-level studies are emerging, all indicating a marked positive effect, especially in very young infants (*12*). This positive effect is further supported by evidence from settings where both maternal and infant immunization programs are implemented, suggesting potential additive benefits in reducing the burden of RSV-related illness (*13*).

During October 2024–March 2025, a large-scale immunization campaign using nirsevimab was launched in Lombardy, Italy, a region of northern Italy with nearly 10 million inhabitants, for infants <12 months of age. We conducted a study to estimate the effects of the campaign on the incidence of emergency department visits (EDVs) and hospital admissions associated with acute LRTI and RSV infection. Because studies conducted so far did not account for season-to-season variability (*6*–*9*), which could result in inaccurate estimates of the effects of RSV immunization, we performed an interrupted time series analysis that incorporated this element to estimate changes in trends before and after the program was implemented.

This project, conducted using routinely collected health data, was deemed by the Directorate General for Health of the Lombardy Region to be public health surveillance (Regional Government Resolution DGR n. XII/3010 of September 9, 2024, concerning the assessment of universal RSV immunization), not research, and was conducted according to applicable regional (Regional Law n. 33/2009, Art. 5 bis) and national law and policy (Italian Legislative Decree n. 196/2003, as amended by Legislative Decree n. 101/2018, Art. 2-sexies). Data processing complied with national and European data protection regulations (General Data Protection Regulation 2016/679).

## Materials and Methods

### Study Design and Population

The study used an interrupted time series design combined with a negative binomial regression model. The analysis covered the period of August 27, 2018 (ISO week 35–2018), through May 11, 2025 (ISO week 19–2025).

The primary study population for evaluating an intermediate intervention outcome consisted of infants <12 months of age residing in the Lombardy region of Italy. All analyses were based on the secondary use of anonymized datasets routinely collected by the Lombardy Region’s Directorate General for Health as part of its public health mandate.

### Data

The regional Emergency Department Syndromic Surveillance system is a new instrument developed by the Prevention Unit of the Lombardy Region’s Directorate General for Health (*14*). Emergency department (ED) records were classified as EDVs if the patient was evaluated in the ED and not admitted; records were classified as hospitalizations if the patient was admitted. Each ED record was classified using codes from the International Classification of Diseases, 9th Revision, Clinical Modification (ICD-9-CM), providing a concise and precise description of the primary diagnosis of the visit. To analyze the performance of the immunization campaign, additional codes for acute LRTI and RSV infection were monitored (Appendix Table 1).

We obtained data on the number of births from the regional demographic registry, which collects and updates vital statistics, including births, deaths, and resident population data. We obtained data on the number of nirsevimab administrations from the regional data flow system, which systematically records individual vaccination events across healthcare settings. Using data on monthly birth cohorts and individual-level vaccination records, we linked each nirsevimab administration to the corresponding child to calculate immunization coverage for birth cohorts during January 2024–March 2025.

### RSV Immunization Campaign

The 2024–25 immunization campaign against RSV infection in the Lombardy region targeted all infants born during January 1, 2024–March 31, 2025. A multicenter approach was used to ensure broad coverage; immunizations were provided through vaccination centers, general pediatricians, hospital pediatric departments, and hospital maternity units.

Beginning October 10, 2024, infants born during January–October 2024 were eligible to receive a single dose of nirsevimab (50 mg or 100 mg according to weight) at vaccination centers or from general pediatricians. Starting November 1, 2024, nirsevimab became available for direct administration at hospital maternity units. Infants who were not immunized at birth were subsequently contacted through vaccination centers or general pediatricians to receive the RSV immunization. Maternal vaccination for RSV was not available in this region or in Italy during the 2024–25 season.

### Statistical Analysis

We aggregated EDVs and hospital admissions into weekly counts and displayed them graphically by ISO week. We defined epidemic seasons as beginning in ISO week 35 of 1 year and ending in ISO week 19 of the following year, capturing the full duration of RSV activity. We stratified descriptive calculations by age group: <1 year and 1–5 years.

To evaluate the effects of the immunization campaign, we conducted interrupted time-series analyses using generalized linear models. The primary impact indicators analyzed in this study included EDVs and hospitalizations for acute LRTI and RSV infection. The intervention period ran from October 10, 2024 (ISO week 41–2024) through March 31, 2025 (ISO week 14–2025).

We trained Poisson and negative binomial regression models using historical data and used them to predict outcomes for the 2024–25 season. Because the seasonal patterns of pre-COVID seasons differ substantially from those after the first waves of COVID-19 in the region, we used data from ISO week 35–2021 to ISO week 41–2024 to train the model. Weekly counts of EDVs and hospitalizations for acute LRTI and RSV infection were used as outcome variables (Appendix). We used the model to generate counterfactual estimates (i.e., the expected number of EDVs and hospitalizations for acute LRTI and RSV infection in the absence of an RSV immunization campaign). We then compared observed cases with those estimates, which were displayed with 95% uncertainty intervals (95% UIs).

To determine whether differences between observed and expected values among infants were caused by a general reduction in RSV circulation rather than the immunization campaign, we compared EDVs and hospitalizations for acute LRTIs and RSV infection in children 1–5 years of age. Those children were not eligible for nirsevimab and served as the comparison group.

We conducted a sensitivity analysis (Appendix) to further corroborate results when excluding diagnoses that could potentially lead to misclassification of EDVs or hospitalizations. We performed all analyses using Python 3.13.0 (https://www.python.org/downloads/release/python-3130) with the pandas, numpy, matplotlib, statsmodels, and patsy packages. We cleaned and managed historical data before December 31, 2024, by using SAS 9.4 (SAS Institute Inc., https://www.sas.com).

## Results

A total of 64,903 doses of nirsevimab were administered by the end of the immunization campaign on March 31, 2025, consisting of 34,913 doses at 100 mg and 29,990 doses at 50 mg. Those doses were given to 61,732 infants out of 77,983 births registered during January 1, 2024–March 31, 2025, resulting in an overall immunization coverage rate of 79.2% (Appendix Table 3).

**Figure 1:**
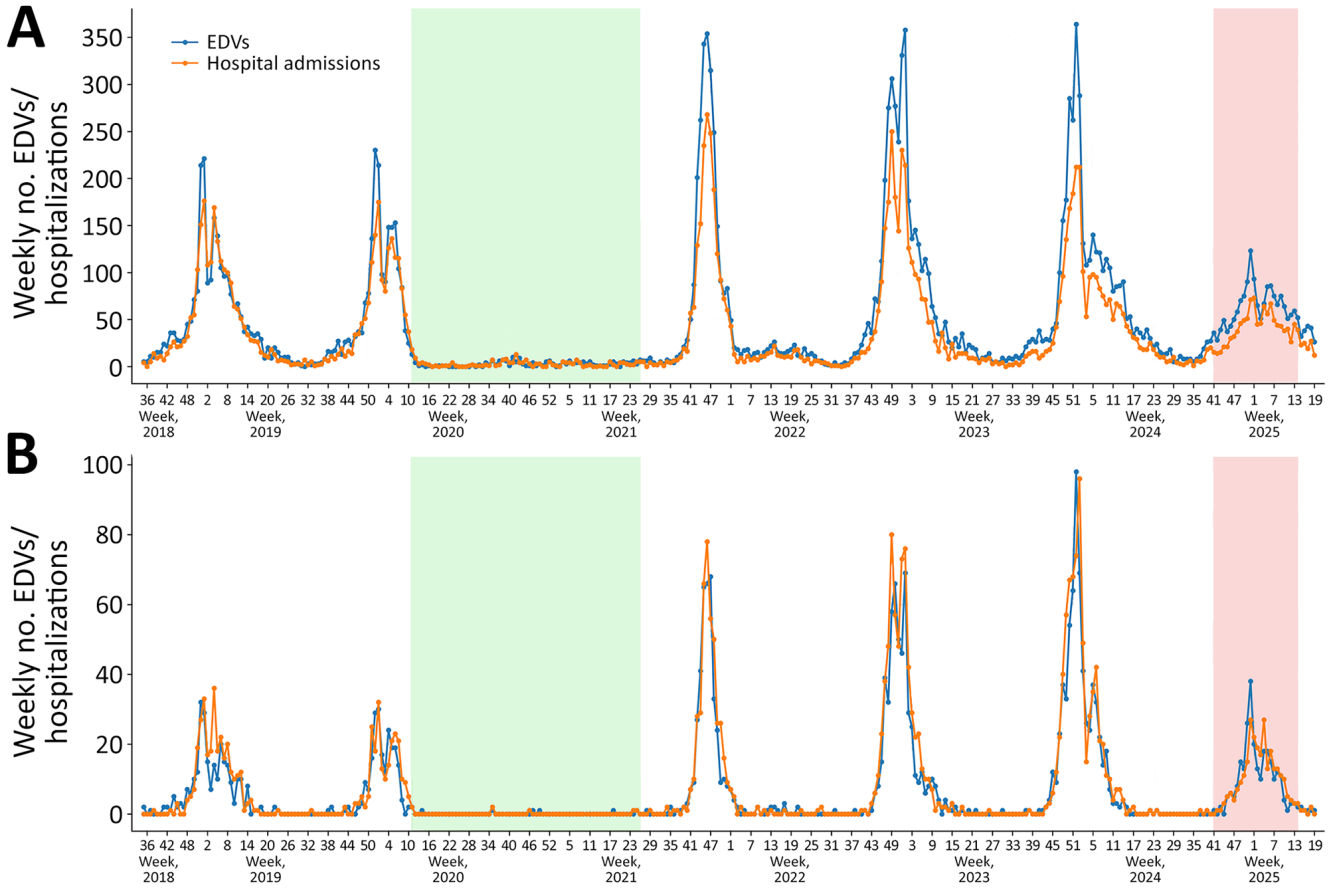
EDVs and hospitalizations among children <12 months of age in study of reduced EDVs and hospitalizations in infants after universal respiratory syncytial virus immunization, Italy, 2024–25. Numbers are shown for ISO week 35–2018 to ISO week 19–2025 for lower respiratory tract infection (A) and respiratory syncytial virus infections (B). Green shading indicates the period during which public health and social measures were implemented in response to the initial waves of the COVID-19 pandemic in the region. Red shading marks the prophylaxis period during which nirsevimab was administered. EDV, emergency department visit.

**Figure 2:**
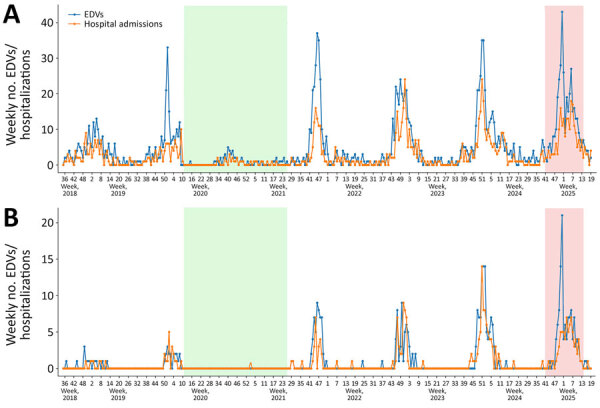
EDVs and hospitalizations in children 1–5 years of age in study of reduced EDVs and hospitalizations in infants after universal respiratory syncytial virus immunization, Italy, 2024–25. Numbers are shown for ISO week 35–2018 to ISO week 19–2025 for lower respiratory tract infection (A) and respiratory syncytial virus infections (B). Green shading indicates the period during which public health and social measures were implemented in response to the initial waves of the COVID-19 pandemic in the region. Red shading marks the prophylaxis period during which nirsevimab was administered. EDV, emergency department visit.

We observed pronounced seasonal trends across both age groups in all winter seasons, except for 2020–21, when public health and social measures were widely implemented in the Lombardy Region in response to the initial waves of the COVID-19 pandemic (Figures 1, 2). Epidemic waves after this period exhibited substantially higher peaks than did pre-COVID seasons (i.e., 2018–19 and 2019–20); patterns of EDVs and hospital admissions were increasingly delayed, and most cases occurred among infants <12 months of age (Figure 1).

In the postpandemic seasons (2021–22, 2022–23, and 2023–24), peaks in EDVs for acute LRTIs and RSV infection among children <12 months of age were typically observed from ISO week 46 (2021–22 season) to ISO week 1 (2022–23 season); an average of 359 (range 354–364) visits for acute LRTIs and 78 (range 68–98) visits for RSV infections occurred (Table 1; Appendix Table 4). Seasonal peaks in both EDVs and hospital admissions for acute LRTI in that age group were lower in prepandemic seasons but though still higher than in 2024–25. For RSV infection, however, the 2024–25 season peak was similar to prepandemic levels; 38 EDVs (vs. 30–32 prepandemic) and 27 hospital admissions (vs. 32–36 prepandemic) were recorded (Appendix Table 4).

**Table 1:** EDVs and hospitalizations for lower respiratory tract infection at seasonal peak week among children in study of reduced EDVs and hospitalizations in infants after universal respiratory syncytial virus immunization, Italy, 2024–25*

Winter season	Children <12 mo			Children 1–5 y	
	EDVs	Hospital admission		EDVs	Hospital admission
2018–19	221 (week 1)	176 (week 1)		13 (week 5)	9 (week 50)
2019–20	230 (week 52)	175 (week 1)		33 (week 52)	10 (week 9)
2020–21	10 (week 41)	13 (week 42)		5 (week 40)	3 (week 41)
2021–22	354 (week 46)	268 (week 46)		37 (week 46)	16 (week 45)
2022–23	358 (week 1)	250 (week 49)		24 (week 49)	24 (week 52)
2023–24	364 (week 52)	212 (week 52)		35 (week 51)	24 (week 51)
2024–25	123 (week 52)	73 (week 1)		43 (week 52)	18 (week 6)

*Numbers shown are for winter seasons from 2018–19 to 2024–25. The ISO week in which the peak was reached is indicated in parentheses. EDV, emergency department visit.

In the same ISO weeks in which EDVs for acute LRTI peaked across seasons among infants <12 months of age, a peak was also recorded among children 1–5 years of age (i.e., between ISO weeks 46 and 51); an average of 32 (range 24–37) EDVs for acute LRTI was recorded (Table 1). Unlike the pattern observed in children <12 months of age, the 2024–25 seasonal peak among children 1–5 years of age reached similar numbers of EDVs and hospital admissions for acute LRTI to the numbers seen in post-COVID seasons (Table 1; Figure 2).

**Figure 3:**
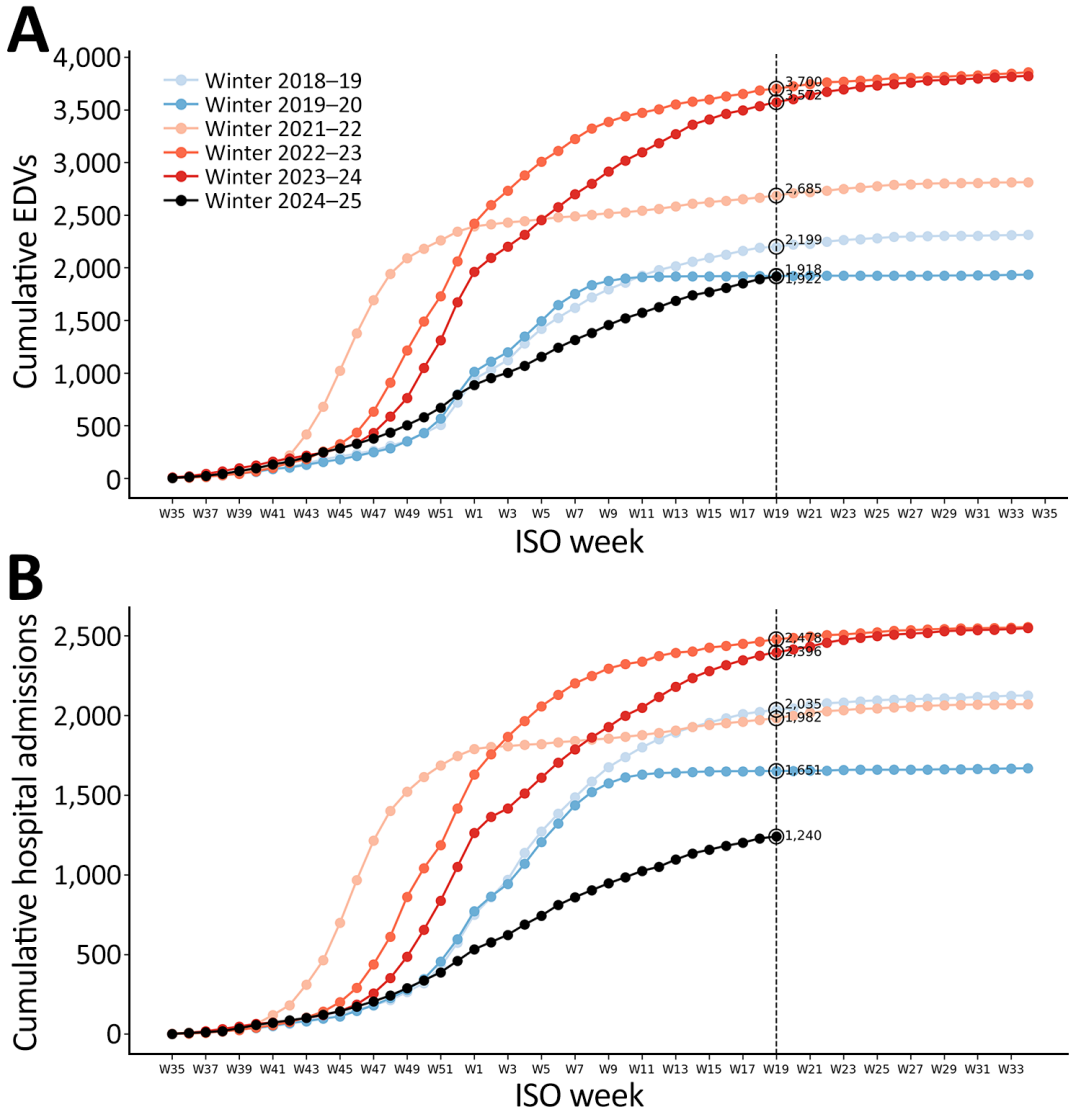
Comparison of cumulative EDVs (A) and hospitalizations (B) for lower respiratory tract infections among children <12 months of age from winter season 2018–19 to 2024–25 in study of reduced EDVs and hospitalizations in infants after universal respiratory syncytial virus immunization, Italy. To enable comparison between different epidemic years, a season was defined as the period going from the 35th week of 1 year to the end of the 34th week of the following year, covering the entire winter epidemic and accounting for shifts in its onset and conclusion. Pre-COVID and post-COVID seasons are colored in different shades of blue and red. EDV, emergency department visit.

When comparing the cumulative number of EDVs and hospital admissions for acute LRTI across different winter seasons among children <12 months of age, the lowest counts were recorded in the 2024–25 season: 1,918 EDVs and 1,240 hospitalizations. The cumulative number of EDVs for acute LRTI was markedly higher in postpandemic seasons than in prepandemic seasons (Figure 3). Similar patterns were observed for RSV infection (Appendix Figure 1).

During the 2021–22 winter season, EDVs and hospitalizations for acute LRTIs increased among infants <12 months of age, resulting in 2,685 EDVs and 1,982 hospital admissions (Figure 3). Those numbers rose further in the 2022–23 and 2023–24 seasons, reaching 3,700 EDVs in 2022–23 and 3,572 EDVs in 2023–24.

In the 2024–25 season, only 1,918 EDVs for acute LRTI were recorded among children <12 months of age (Table 2; Appendix Figure 3, panel A), marking the lowest number of EDVs for the condition across all winter seasons studied, including both prepandemic and postpandemic periods. Compared with 3,700 EDVs in the 2022–23 season and 3,572 EDVs in the 2023–24 season, that difference represents a decline of >50%. Even more pronounced reductions were observed in hospital admissions for acute LRTI, which fell to 1,240 during the 2024–25 season, compared with 2,396 in the 2023–24 season (a 48% reduction) and 2,478 in the 2022–23 season (a 50% reduction) (Figure 3, panel B). Although the peak of EDVs and hospital admissions for RSV infection in the 2024–25 season was similar to that observed in prepandemic seasons (Appendix Table 4), cumulative counts in the postpandemic seasons (from 2021–2024) were substantially higher (Appendix Figure 1). Compared with the 2022–23 season, the 2024–25 season showed a 59% reduction in EDVs (from 644 to 265) and a 61% reduction in hospital admissions (from 704 to 273) among children <12 months of age.

**Table 2:** Impact of universal RSV immunization on EDVs and hospitalization in children <12 months of age and those 1–5 years of age, Italy, 2024–25*

Age cohort	Indicator activity, winter 2024–25			Difference in indicator activity	
	Predicted (95% UI)	Observed		Absolute change	% Change (95% UI)
Children <12 mo					
LRTI					
EDVs	3,347 (3,067–3,627)	1,918		−1,429	−42.7 (−48.1 to −37.3)
Hospitalizations	2,316 (2,120–2,513)	1,240		−1,076	−46.5 (−51.9 to −41.0)
RSV infection					
EDVs	523 (456–590)	265		−258	−49.3 (−58.2 to −40.4)
Hospitalizations	607 (523–691)	273		−334	−55.0 (−63.2 to −46.8)
Children 1–5 y					
LRTI					
EDVs	287 (262–312)	393		106	36.9 (18.9–55.0)
Hospitalizations	160 (145–176)	225		65	40.6 (17.6–63.6)
RSV infection					
EDVs	68 (55–80)	116		48	70.6 (26.5–114.7)
Hospitalizations	46 (36–55)	75		29	63.0 (13.0–113.0)	

*EDV, emergency department visit; LRTI, lower respiratory tract infection; RSV, respiratory syncytial virus; UI, uncertainty interval.

### Interrupted Time Series Analysis

**Figure 4:**
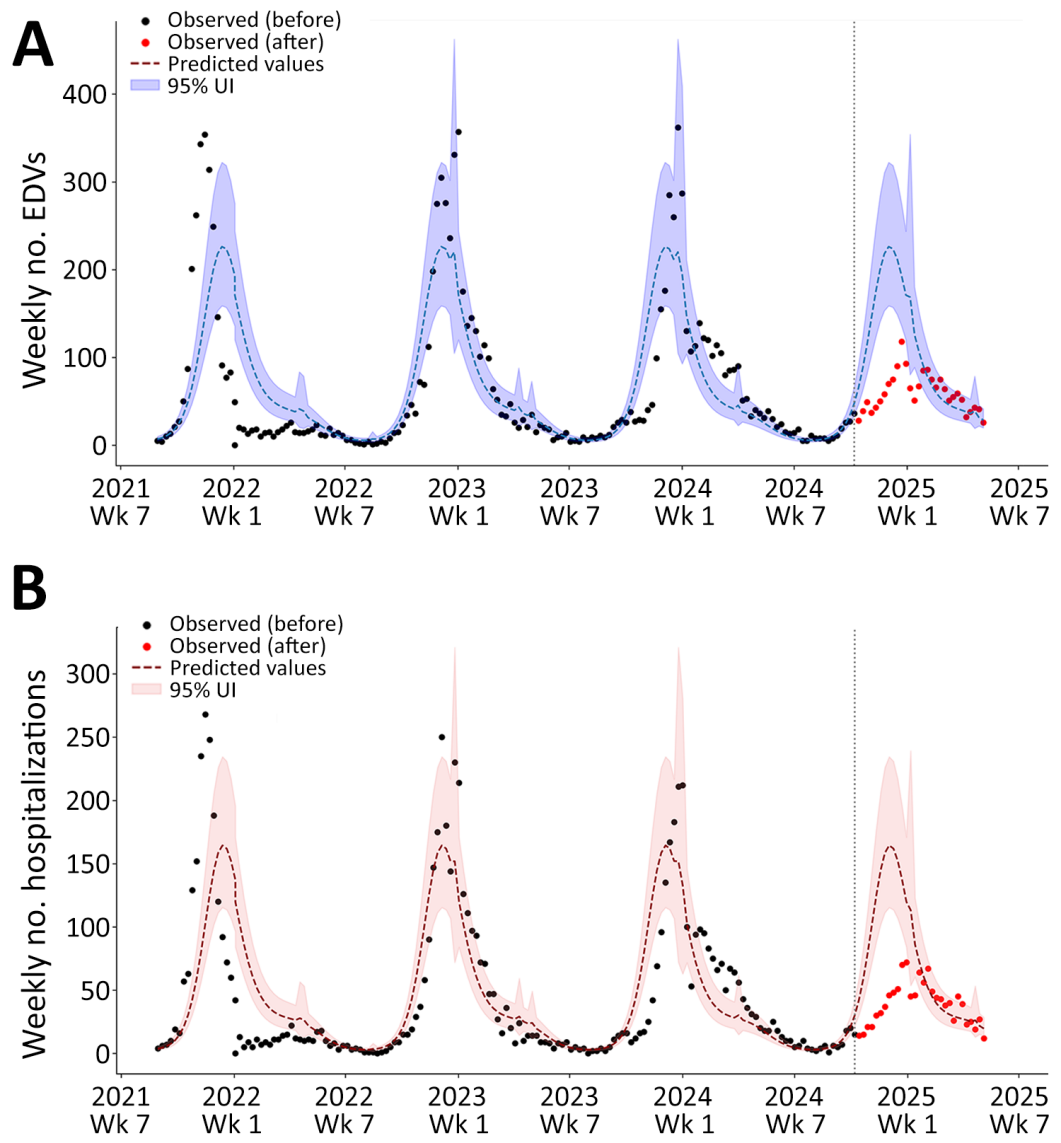
Interrupted time series analysis of EDVs (A) and hospitalizations (B) for lower respiratory tract infections among infants <12 months of age in study of reduced EDVs and hospitalizations in infants after universal respiratory syncytial virus immunization, Italy, 2024–25. Numbers are shown for ISO week 35–2021 to ISO week 19–2025. A negative binomial model incorporating a Fourier parameter was trained using historic data before the launch of the universal respiratory syncytial virus immunization program (before October 10, 2024; vertical dashed lines indicate launch date) and was then used to compute predicted values and 95% UIs. Those values are displayed against the observed weekly counts of EDVs and hospital admissions for lower respiratory tract infections. EDV, emergency department visit; UI, uncertainty interval.

**Figure 5:**
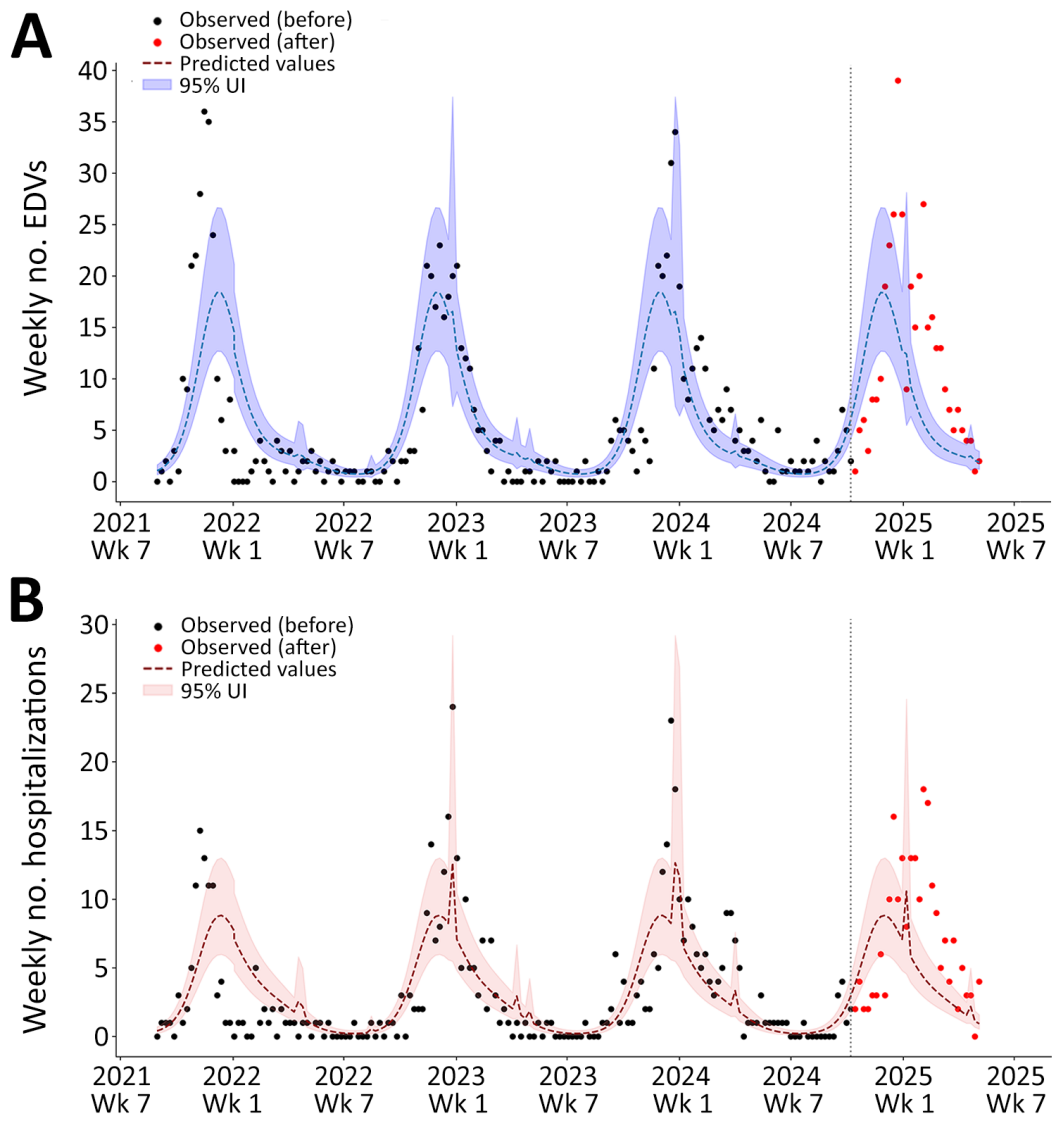
Interrupted time series analysis of EDVs (A) and hospitalizations (B) for lower respiratory tract infections among children 1–5 years of age in study of reduced EDVs and hospitalizations in infants after universal respiratory syncytial virus immunization, Italy, 2024–25. Numbers are shown for ISO week 35–2021 to ISO week 19–2025. A negative binomial model incorporating a Fourier parameter was trained using historic data before the launch of the universal respiratory syncytial virus immunization program (before October 10, 2024; vertical dashed lines indicate launch date) and was then used to compute predicted values and 95% UIs. Those values are displayed against the observed weekly counts of EDVs (and hospital admissions for lower respiratory tract infections. EDV, emergency department visit; UI, uncertainty interval.

Regression modeling suggested that EDVs and hospitalizations for acute LRTI and RSV infection plummeted among children <12 months of age during the 2024–25 season (Table 2; Figure 4; Appendix Figure 2). We observed comparable declines for LRTI and RSV infections among the same age group, both in EDVs (−42.7% for LRTIs and −49.3% for RSV infection) and hospital admissions (−46.5% for LRTIs and −55.0% for RSV infection). By contrast, children 1–5 years of age, who were not the target population of the immunization campaign, showed an opposite trend (Figure 5; Appendix Figure 3). Although those children had lower absolute numbers of both EDVs and hospitalization than infants <12 months of age, their indicator activity increased during the 2024–25 season. Specifically, predicted EDVs for RSV infection were 68 (95% UI 55–80), whereas observed visits were 116, a 70.6% (95% UI 26.5–114.7) increase. Those values exceeded those observed in previous post-COVID seasons (2021–22 to 2023–24), confirming persistent RSV circulation among children 1–5 years of age. Similarly, predicted hospital admissions for RSV infection were 46 (95% UI 36–55), compared with 75 observed admissions, a 63.0% (95% UI 13.0– 113.0) increase.

### Sensitivity Analysis

Diagnoses of ICD-9-CM code 07.96 (i.e., RSV infection) accounted for 0.2% of EDVs and hospitalizations for acute LRTIs among children <12 months of age and accounted for 2.0% of EDVs and 0.8% of hospital admissions among children 1–5 years of age (Appendix Table 5, Figures 4, 5). Excluding that diagnosis code from the acute LRTI definition produced only minor differences in the estimated impact of universal RSV immunization, underscoring the robustness of the analysis (Appendix Table 6).

## Discussion

We present the results of an evaluation of the effectiveness of a large-scale nirsevimab immunization campaign for infants <12 months of age. The campaign aimed to reduce EDVs and hospital admissions for acute LRTI and RSV infection in the Lombardy region of northern Italy.

We observed a significant overall decline in EDVs and hospitalizations for acute LRTI and RSV infection among infants <12 months of age; we observed comparable percentage reductions in both EDVs and hospitalizations relative to both historical trends and model-predicted values. Starting November 2024, most infants were immunized at birth, ensuring timely protection. This campaign likely contributed to the observed impact, as opposed to outpatient-based strategies that rely on postdischarge follow-up. However, this decline is unlikely attributable to reduced or interrupted RSV circulation during the 2024–25 season, given that the trends observed in the 1–5–year age group remained consistent with or exceeded historical and predicted levels. The observed reduction in RSV-related outcomes in infants was not mirrored in children 1–5 years of age, suggesting that the intervention likely prevented disease among infants, not that levels of RSV circulation in the population were reduced.

A recent analysis modeled different immunization and vaccination scenarios in the Lombardy region using data collected before the RSV campaign was implemented to inform regional public health policies (*15*). That analysis estimated a considerable reduction in the number of hospitalizations caused by RSV: a 44% decrease with an estimated immunization coverage rate of 70% and a 60% decrease with an estimated immunization coverage rate of 95%. Those findings are consistent with the results of our analysis, which found that EDVs associated with RSV hospitalization decreased by 55% in the 2024–25 season. The previous study further predicted that the intervention would have had limited effects on reducing overall RSV circulation, because infants contribute relatively little to RSV community transmission (*15*). Our study corroborated that finding; an opposing trend was observed in children 1–5 years of age, supporting the use of this age group as a comparison population for deriving counterfactual estimates.

The effect of the RSV immunization campaign observed in our study is further supported by effectiveness estimates derived from a density case–control study (*16*), which were subsequently applied to the eligible infant population in Spain to estimate the national impact (*17*). That extrapolation suggests that, for every 1,000 infants immunized with the mAb, 14.7 cases of RSV-associated acute LRTIs are prevented. Likewise, we observed that, for every 1,000 mAb doses administered, 22.0 EDVs (1,429/64,903) and 16.6 hospital admissions (1,076/64,903) for acute LRTI were prevented.

Similar findings have been reported in Luxembourg (*10*), where an estimated coverage of 84% was achieved in maternity wards across the country’s 4 hospitals (≈1,524 births), leading to a 69% reduction in hospitalizations among infants <6 months of age with laboratory-confirmed RSV infection. A notable increase in the mean age of RSV-associated hospitalizations was observed in that study for 2024–25 compared with 2022–23; the mean age shifted from 7.8 months in 2022–23 to 14.4 months in 2024–25. That shift in age distribution might partly explain the differences observed in our study, where EDVs and hospitalizations were considered in infants <12 months of age, in addition to potential variations in immunization coverage.

As expected, no surge in EDVs or hospitalizations for acute LRTI or RSV infection was observed during the rigorous implementation of public health and social measures in the initial phase of the pandemic in 2020 and 2021. Compared with previous epidemics, the postpandemic periods recorded the highest number of EDVs and hospital admissions. That phenomenon might be attributed to waning immunity against RSV as a result of the stringent public health and social measures implemented to curb the surge of COVID-19 cases. Those measures ultimately reduced the circulation of several respiratory viruses, including RSV (*18*,*19*).

Our analysis has several limitations arising from the reliance on administrative anonymized datasets rather than laboratory RSV test results. First, testing intensity for RSV might have varied over the study period, particularly with increased diagnostic efforts in recent years (Appendix Table 5). Those differences might have led to greater detection of mild or non-LRTI RSV, affecting comparability across seasons. To mitigate that possibility, we adopted a broader case definition for acute LRTI that relied less on microbiological confirmation. Sensitivity analyses further suggested that non-LRTI RSV infections had only a marginal effect on the model estimates. Second, the datasets we used were not originally designed to identify individual cases. We made efforts to remove duplicates from EDVs and hospitalization data using a set of criteria, but that process was further complicated by the impossibility of ascertaining patients’ exposure to the intervention under assessment. Therefore, an unimmunized infant might have visited different hospitals on different days, resulting in multiple counts without the possibility of deduplication. Third, because the case definition used for acute LRTI includes ICD-9-CM code 079.6, some episodes might include upper respiratory tract infections, leading to potential overestimation of RSV-related acute LRTI episodes. However, on the basis of the sensitivity analysis we conducted, that factor did not affect impact estimates when we removed diagnoses coded 079.6 from the case definition of acute LRTIs. Fourth, although most studies have focused on infants <6 months of age, we were unable to replicate that cutoff because of the nature of the ED data and the fact that the target population of the universal immunization campaign included all infants born during 2024–March 2025. Although the datasets used in our analysis encompass both date of birth and age group, only age group is a mandatory variable, because it is either directly recorded during the EDV or derived from date of birth. For children, the available age categories distinguish only between those <12 months of age and those 1–5 years of age. Consequently, our analysis was constrained to the infants <12 months of age, rather than infants <6 months of age. Fifth, the 2024–25 RSV season might not have been fully captured within the analysis period, potentially leading to underestimation of RSV-related outcomes for that season. That factor should be considered when comparing with earlier seasons that were fully observed. Last, because of the nature of the dataset used, persons residing outside the region who might have sought care at 1 of the ≈100 hospitals could not be excluded. That limitation is likely to have a minimal impact at the regional level, but it could be more relevant in areas near the borders of other regions in Italy or near Switzerland, where cross-border healthcare use could occur in both directions.

This study explored the effects of nirsevimab administration in a large territory with a population of ≈10 million persons and ≈64,500 births in 2024 (*20*) (63,053 of whom lived in the region), focusing on the number of EDVs and hospital admissions both for acute LRTIs and RSV infection among infants <12 months of age. We compared results with historical trends as well as to a comparison group to elucidate the effect of the immunization campaign. We found that use of nirsevimab for passive immunization of newborns and children <12 months of age, achieving an immunization coverage rate of ≈79% across the Lombardy region of Italy, resulted in a substantial reduction in EDVs and hospital admissions for acute LRTI and RSV infection. Further studies will be conducted upon the conclusion of the RSV epidemic to estimate the cost-effectiveness of the immunization campaign implemented. Our results, combined with those future analyses, will help inform policy decisions for future immunization programs.

Appendix Additional information about reduced emergency department visits and hospitalizations in infants after universal respiratory syncytial virus immunization, Italy, 2024–25

## References

[1] Blau DM, Baillie VL, Els T, Mahtab S, Mutevedzi P, Keita AM, et al.; CHAMPS Consortium. CHAMPS Consortium. Deaths attributed to respiratory syncytial virus in young children in high-mortality rate settings: report from Child Health and Mortality Prevention Surveillance (CHAMPS). Clin Infect Dis. 2021;73(Suppl_3):S218–28. 34472577 10.1093/cid/ciab509 PMC8411256

[2] European Medicines Agency. Beyfortus (nirsevimab) [cited 2025 May 23]. https://www.ema.europa.eu/en/medicines/human/EPAR/beyfortus

[3] Hammitt LL, Dagan R, Yuan Y, Baca Cots M, Bosheva M, Madhi SA, et al.; MELODY Study Group. Nirsevimab for prevention of RSV in healthy late-preterm and term infants. N Engl J Med. 2022;386:837–46. 35235726 10.1056/NEJMoa2110275

[4] Griffin MP, Yuan Y, Takas T, Domachowske JB, Madhi SA, Manzoni P, et al.; Nirsevimab Study Group. Single-dose nirsevimab for prevention of RSV in preterm infants. N Engl J Med. 2020;383:415–25. 32726528 10.1056/NEJMoa1913556

[5] Drysdale SB, Cathie K, Flamein F, Knuf M, Collins AM, Hill HC, et al.; HARMONIE Study Group. Nirsevimab for prevention of hospitalizations due to RSV in infants. N Engl J Med. 2023;389:2425–35. 38157500 10.1056/NEJMoa2309189

[6] Moline HL, Tannis A, Toepfer AP, Williams JV, Boom JA, Englund JA, et al.; New Vaccine Surveillance Network Product Effectiveness Collaborators. Early estimate of nirsevimab effectiveness for prevention of respiratory syncytial virus-associated hospitalization among infants entering their first respiratory syncytial virus season—New Vaccine Surveillance Network, October 2023–February 2024. MMWR Morb Mortal Wkly Rep. 2024;73:209–14. 38457312 10.15585/mmwr.mm7309a4 PMC10932582

[7] Xu H, Aparicio C, Wats A, Araujo BL, Pitzer VE, Warren JL, et al. Estimated effectiveness of nirsevimab against respiratory syncytial virus. JAMA Netw Open. 2025;8:e250380. 40063022 10.1001/jamanetworkopen.2025.0380 PMC11894488

[8] Ares-Gómez S, Mallah N, Santiago-Pérez MI, Pardo-Seco J, Pérez-Martínez O, Otero-Barrós MT, et al.; NIRSE-GAL study group. Effectiveness and impact of universal prophylaxis with nirsevimab in infants against hospitalisation for respiratory syncytial virus in Galicia, Spain: initial results of a population-based longitudinal study. Lancet Infect Dis. 2024;24:817–28. 38701823 10.1016/S1473-3099(24)00215-9

[9] Sumsuzzman DM, Wang Z, Langley JM, Moghadas SM. Real-world effectiveness of nirsevimab against respiratory syncytial virus disease in infants: a systematic review and meta-analysis. Lancet Child Adolesc Health. 2025;9:393–403. 40319903 10.1016/S2352-4642(25)00093-8

[10] Ernst C, Bejko D, Gaasch L, Hannelas E, Kahn I, Pierron C, et al. Impact of nirsevimab prophylaxis on paediatric respiratory syncytial virus (RSV)-related hospitalisations during the initial 2023/24 season in Luxembourg. Euro Surveill. 2024;29:2400033. 38275017 10.2807/1560-7917.ES.2024.29.4.2400033 PMC10986653

[11] López-Lacort M, Muñoz-Quiles C, Mira-Iglesias A, López-Labrador FX, Mengual-Chuliá B, Fernández-García C, et al. Early estimates of nirsevimab immunoprophylaxis effectiveness against hospital admission for respiratory syncytial virus lower respiratory tract infections in infants, Spain, October 2023 to January 2024. Euro Surveill. 2024;29:2400046. 38333937 10.2807/1560-7917.ES.2024.29.6.2400046 PMC10853977

[12] Perramon-Malavez A, Buonsenso D, Morello R, Coma E, Foster S, Leonard P, et al. Real-world impact of nirsevimab immunisation against respiratory disease on emergency department attendances and admissions among infants: a multinational retrospective analysis. Lancet Reg Health Eur. 2025;55:101334. 40950943 10.1016/j.lanepe.2025.101334 PMC12426819

[13] Patton ME, Moline HL, Whitaker M, Tannis A, Pham H, Toepfer AP, et al. Interim evaluation of respiratory syncytial virus hospitalization rates among infants and young children after introduction of respiratory syncytial virus prevention products—United States, October 2024–February 2025. MMWR Morb Mortal Wkly Rep. 2025;74:273–81. 40338822 10.15585/mmwr.mm7416a1 PMC12061057

[14] Villa S, Maffeo M, Maistrello M, Bagarella G, Porrello VN, Morani F, et al. Increased pneumonia-related emergency department visits, northern Italy. Emerg Infect Dis. 2025;31:1057–9. 40249950 10.3201/eid3105.241790 PMC12044229

[15] Menegale F, Vezzosi L, Tirani M, Scarioni S, Odelli S, Morani F, et al. Impact of routine prophylaxis with monoclonal antibodies and maternal immunisation to prevent respiratory syncytial virus hospitalisations, Lombardy region, Italy, 2024/25 season. Euro Surveill. 2025;30:2400637. 40211969 10.2807/1560-7917.ES.2025.30.14.2400637 PMC11987492

[16] Núñez O, Olmedo C, Moreno-Perez D, Lorusso N, Fernández Martínez S, Pastor Villalba PE, et al.; Nirsevimab Effectiveness Study Collaborators. Effectiveness of catch-up and at-birth nirsevimab immunisation against RSV hospital admission in the first year of life: a population-based case-control study, Spain, 2023/24 season. Euro Surveill. 2025;30:2400596. 39916606 10.2807/1560-7917.ES.2025.30.5.2400596 PMC11803741

[17] Pastor-Barriuso R, Núñez O, Monge S; Nirsevimab Effectiveness Study Collaborators. Infants needed to immunise with nirsevimab to prevent one RSV hospitalisation, Spain, 2023/24 season. Euro Surveill. 2025;30:2500040. 39949320 10.2807/1560-7917.ES.2025.30.6.2500040 PMC11914966

[18] Abu-Raya B, Viñeta Paramo M, Reicherz F, Lavoie PM. Why has the epidemiology of RSV changed during the COVID-19 pandemic? EClinicalMedicine. 2023;61:102089. 37483545 10.1016/j.eclinm.2023.102089 PMC10359735

[19] Ang HJ, Menegale F, Preziosi G, Pariani E, Migliari M, Pellegrinelli L, et al. Reconstructing the impact of COVID-19 on the immunity gap and transmission of respiratory syncytial virus in Lombardy, Italy. EBioMedicine. 2023;95:104745. 37566927 10.1016/j.ebiom.2023.104745 PMC10432612

[20] Istituto Nazionale di Statistica. Indicatori demografici–Anno 2024 [cited 2025 May 30]. https://www.istat.it/comunicato-stampa/indicatori-demografici-anno-2024

