# Supplementary material for: Reduced Emergency Department Visits and Hospitalizations in Infants after Universal Respiratory Syncytial Virus Immunization, Italy, 2024–25
Source: Emerg Infect Dis. 2026 Jan;32(1):45–54. doi: 10.3201/eid3201.250870 (PMC12870101; doi:10.3201/eid3201.250870)
Supplement: Appendix — Additional information about reduced emergency department visits and hospitalizations in infants after universal respiratory syncytial virus immunization, Italy, 2024–25 [file 25-0870-Techapp-s1.pdf]

Article DOI: https://doi.org/10.3201/eid3201.250870

EID cannot ensure accessibility for supplementary materials supplied by authors. Readers who have difficulty accessing supplementary content should contact the authors for assistance.

Reduced Emergency Department Visits and Hospitalizations in Infants after Universal Respiratory Syncytial Virus Immunization, Italy, 2024–25

Appendix

Supplementary Methods

De-duplication process

Due to the anonymous nature of the datasets used in this study, it was not possible to ascertain how many times a subject visited either the same or another ED. However, since the datasets used are updated in real time, and potential delays may result in the duplication of cases, records were de-duplicated using the date and place of birth, the date and time of ED visit (EDV), and sex. This procedure allowed us to exclude cases in which a patient was admitted to hospital after an ED visit, thereby avoiding double counting the same episode.

Modeling strategy

The dataset was divided into four data frames by age group (children under 12 months and children aged 1–5 years) and diagnosis (acute LRTIs and RSV infections). Each was further split into training data (ISO weeks 35–2021 to before week 41–2024) and testing data (ISO weeks 42–2024 to 19–2025).

Predictions assuming the absence of an RSV immunization campaign were generated for the test period using Poisson and Negative Binomial regression models for count data. Both models used a logarithmic (log) link function and incorporated harmonic terms based on Fourier transformations (sine and cosine functions), to better capture seasonal patterns. To accommodate asymmetric or complex seasonality, the Fourier series included up to the second harmonic. To account for potential weekly variation due to holidays, a binary variable was included to indicate the presence of a public holiday in the week. The structure of the modeling strategy is outlined below.

$$\log(\mu_t) = \beta_0 + \beta_1 \cdot Holiday_t + \Sigma_{k=1}^{K} \left[\alpha_k \cdot \sin\left(\frac{2\pi kt}{52}\right) + \gamma_k \cdot \cos\left(\frac{2\pi kt}{52}\right) \right]$$

In this expression, *t* represents time (in ISO week); *k* refers to the order of the harmonic used to capture seasonal variation (e.g., *k* = 1 or 2); and *K* represents the total number of harmonics included. Fourier terms were included up to the second harmonic (*K* = 2) to model complex seasonal patterns.

Because Poisson regression often has overdispersion, which can bias parameter estimates, model fit was compared using the overdispersion ratio and the Akaike information criterion (AIC). The first is used to assess whether the variability in the data exceeds what is expected under a Poisson distribution. A ratio close to 1 is generally considered acceptable, meaning that that the model's variance assumptions are appropriate, whereas values significantly greater than 1 suggest overdispersion, indicating that a negative binomial model may be more appropriate. The AIC is a measure of the model quality, balancing the goodness of fit with model complexity. Lower AIC values indicate a better-fitting model. A Negative Binomial model, which explicitly models overdispersion via a dispersion parameter, showed lower AIC values and was therefore selected for the analyses.

The predicted seasonal total was then computed as

$$\hat{P} = \sum_{t=1}^{T} \widehat{\mu_t}$$

where $\widehat{\mu_t}$ is the predicted count at week t obtained from the model. The standard standard error ($SE(\hat{P})$) of the aggregated total was computed, assuming normality and independence across weeks.

$$SE(\hat{P}) = \sqrt{\sum_{t=1}^{T} \left(\frac{\widehat{\mu_t}(upper) - \widehat{\mu_t}(lower)}{2 \cdot 1.96} \right)^2}$$

The 95% uncertainty interval (UI) for the total predicted counts was defined as:

$$95\%UI(\hat{P}) = \hat{P} \pm 1.96 \cdot SE(\hat{P})$$

The absolute difference (D) was defined as the observed total minus the predicted total

$$D = O - \hat{P}$$

with standard error computed as follows

$$SE(D) = \sqrt{SE(\hat{P})^2 + SE(O)^2}$$

where the standard error of the observed total was approximate assuming a Poisson distribution.

$$SE(O) = \sqrt{O}$$

The percentage change (or relative difference, RD) was derived using the following formulas, computing the absolute difference from observed (O) and pedicted ($\hat{P}$) counts from the model, divided by predicted values.

$$RD = \frac{O - \hat{P}}{\hat{P}} \times 100$$

Under the delta method (1) approximation and assuming independence between observed and predicted counts (i.e., covariance = 0), the variance of RD is:

$$Var(RD) = \frac{SE(O)^2}{\hat{P}^2} + \frac{O^2 \cdot SE(\hat{P})^2}{\hat{P}^4}$$

Then, the standard error of RD is:

$$SE(RD) = \sqrt{Var(RD)}$$

And the 95%UI calculated as follows

$$95\%UI(RD) = RD \pm 1.96 \cdot SE(RD)$$

The delta method is a "first-order" approximation based on a linear Taylor expansion, which provides a stable and computationally efficient estimate of uncertainty. While the bootstrap is a "second-order" approximation that can account for nonlinearity and empirically capture sampling variability, in our context, with low weekly counts and some zeros, the delta-method approach produces more stable and interpretable UIs.

Sensitivity Analysis

A revised definition of acute LRTIs was used by excluding ICD-9-CM code 07.96 (i.e., infection by respiratory syncytial virus), which might lead to potential misclassification of acute LRTIs episodes, both EDVs and hospital admissions.

As shown in Appendix Figure 4 and Appendix Figure 5, most episodes of EDVs and hospital admissions of acute LRTIs have a very small number of diagnoses coded as 07.96. Among children under 12 months, a total of 36 EDVs (86.1% during post-COVID seasons) and 19 hospital admissions (73.7% during post-COVID seasons) with a diagnosis coded 07.96 were registered

during the study period. Similar absolute counts were observed among children aged 1–5 years, as described in Appendix Table 5.

Most of episodes were attributed to code 466.19 (i.e., Other bronchiolitis), ranging from 82.9% to 88.8% (excluding 2020–2021 season) among EDVs in children aged <12 months, and 67.7% to 91.6% among those aged 1–5 years. Diagnoses coded 466.11 (i.e., RSV bronchiolitis) were the second most frequent among acute LRTI EDVs ranging from 10.9% to 16.6% (excluding 2020–2021 season) among children <12 months, and 7.9% to 26.0% in children aged 1–5 years.

Similar patterns were observed for hospital admissions, with pneumonia due to RSV (ICD-9-CM code 480.1) being diagnosed among children aged 1–5 years (ranging from 1.8% to 2.8%), particularly during the post-COVID-19 seasons (Appendix Table 5).

As in the main analysis, after excluding diagnoses coded 07.96 and applied the case definition for acute LRTIs, the dataset was divided into two data frames by age group (children under 12 months and children aged 1–5 years). Each was further split into two sets of data, one used for training the model (from ISO weeks 35–2021 to before 41–2024) and a second set to test the model (from ISO weeks 42–2024 to 19–2025). Post-diagnostic analyses using AIC and overdispersion ratios were conducted, as described in the **Supplementary Methods**, to identify the appropriate model between Poisson or negative binomial regressions, incorporating Fourier terms with components up to the second harmonic.

The impact on RSV immunization among children aged <12 months, when excluding code 07.96 from EDVs and hospitalizations for acute LRTIs, did not change (Appendix Table 6). Marginal differences (<2%) could only be observed among children of 1–5 years for which both EDVs and hospitalizations increased in season 2024–2025 (Table 2 in main article; Appendix Table 6). Specifically, EDVs increased from 36.9% (95%UI: 20.8 to 53.1) to 41.1% (95%UI:20.1 to 62.1) when excluding diagnoses coded 07.96. Likewise, hospital admissions increased from 40.6% (95%UI: 19.8 to 61.5) to 41.1% (95%UI: 20.1 to 62.1).

References

1. Zhang F, Wagner AK, Soumerai SB, Ross-Degnan D. Methods for estimating confidence intervals in interrupted time series analyses of health interventions. J Clin Epidemiol. 2009;62:143–8. PubMed

Appendix Table 1. Codes and definitions of diagnoses from Emergency Department visit records used for syndromic surveillance.

ICD-9-CM	Definition	Acute LRTI	RSV infection
079.6	Respiratory syncytial virus	Yes	Yes
466.1	Acute bronchiolitis	Yes	No
466.11	Acute bronchiolitis due to RSV	Yes	Yes
466.19	Acute bronchiolitis due to other infectious organisms	Yes	No
480.1	Pneumonia due to RSV	Yes	Yes

ICD-9-CM, International Classification of Diseases, Ninth Revision, Clinical Modification; LRTI, lower respiratory tract infection; RSV, respiratory syncytial virus.

Appendix Table 2. Model comparison of emergency department visits and hospitalizations for acute lower respiratory tract and RSV infection in children <12 mo and 1–5 y*

	POISSON MODEL		NEGATIVE BINOMIAL MODEL
Age cohort	AIC	Overdispersion ratio	AIC
**Children <12 mo**			
LRTI			
EDVs	5252.1	27.8	1522.6
Hospitalizations	3901.1	19.7	1379.9
RSV infection			
EDVs	1452.9	6.9	720.0
Hospitalizations	1476.9	7.1	723.6
**Children 1–5 y**			
LRTI			
EDVs	997.0	3.5	801.9
Hospitalizations	704.4	2.2	632.8
RSV infection			
EDVs	383.3	1.5	314.5
Hospitalizations	324.8	1.2	283.3

*Each outcome (LRTI and RSV) was modeled separately using both Poisson and Negative Binomial regressions. The table reports AIC values and overdispersion ratios for each model. EDV, emergency department visit; LRTI, lower respiratory tract infection; RSV, respiratory syncytial virus; UI, uncertainty interval.

Appendix Table 3. Number of births and corresponding immunization coverages by month of birth

Month	Births	Immunized	Immunization coverage (%)	Cumulative births	Cumulative subjects immunized	Cumulative immunization coverage (%)
Jan-2024	5,479	2,740	50.0	5,479	2,740	50.0
Feb-2024	4,999	2,702	54.1	10,478	5,442	51.9
Mar-2024	5,078	3,027	59.6	15,556	8,469	54.4
Apr-2024	4,789	3,478	72.6	20,345	11,947	58.7
May-2024	5,203	4,086	78.5	25,548	16,033	62.8
Jun-2024	4,979	4,078	81.9	30,527	20,111	65.9
Jul-2024	5,478	4,632	84.6	36,005	24,743	68.7
Aug-2024	5,377	4,620	85.9	41,382	29,363	71.0
Sep-2024	5,362	4,726	88.1	46,744	34,089	72.9
Oct-2024	5,540	4,926	88.9	52,284	39,015	74.6
**Immunization of newborns in maternity wards**				**25,699**	**22,717**	**88.4**
Nov-2024	5,444	4,962	91.1	57,728	43,977	76.2
Dec-2024	5,325	4,827	90.6	63,053	48,804	77.4
Jan-2025	5,353	4,832	90.3	68,406	53,636	78.4
Feb-2025	4,720	4,196	88.9	73,126	57,832	79.1
Mar-2025	4,857	3,900	80.3	77,983	61,732	79.2
**Overall births and immunization coverages**				**77,983**	**61,732**	**79.2**

Appendix Table 4. Number of emergency departments visits and hospitalizations for RSV infection at seasonal peak week among children under 12 mo and those aged 1–5 y in the winter seasons 2018–2019 to 2024–2025*

	Children <12 mo		Children 1–5 y	
Winter Season	EDVs	Hospital admissions	EDVs	Hospital admissions
2018–2019	32 (week 52)	36 (week 04)	3 (week 49)	1 (week 50)
2019–2020	30 (week 01)	32 (week 01)	7 (week 01)	5 (week 01)
2020–2021	1 (week 35)	2 (week 35)	2 (week 29)	1 (week 02)
2021–2022	68 (week 47)	78 (week 46)	12 (week 45)	7 (week 45)
2022–2023	69 (week 01)	80 (week 49)	15 (week 47)	9 (week 51)
2023–2024	98 (week 52)	96 (week 01)	26 (week 51)	14 (week 51)
2024–2025	38 (week 52)	27 (week 52)	26 (week 52)	7 (week 03)

*The ISO week in which the peak was reached is indicated in brackets. EDV, emergency department visit.

Appendix Table 5. Diagnosis codes used for Emergency Department Visits and hospitalizations among infants <12 mo and children 1–5 y across winter seasons, from 2018 to 2025

Age cohort	2018–2019	2019–2020	2020–2021	2021–2022	2022–2023	2023–2024	2024–2025
**Children <12 mo**							
EDVs							
Code 07.96	0 (0.0)	5 (0.3)	0 (0.0)	6 (0.2)	9 (0.2)	11 (0.3)	5 (0.3)
Code 466.1	0 (0.0)	0 (0.0)	0 (0.0)	0 (0.0)	21 (0.5)	9 (0.2)	45 (2.3)
Code 466.11	259 (11.2)	211 (10.9)	6 (2.9)	398 (14.2)	516 (13.4)	633 (16.6)	259 (13.5)
Code 466.19	2053 (88.8)	1718 (88.8)	201 (97.1)	2408 (85.6)	3309 (85.8)	3167 (82.9)	1608 (83.8)
Code 480.1	0 (0.0)	0 (0.0)	0 (0.0)	0 (0.0)	1 (0.0)	2 (0.1)	1 (0.1)
Hospital admissions							
Code 07.96	2 (0.1)	3 (0.2)	0 (0.0)	2 (0.1)	2 (0.1)	7 (0.3)	3 (0.2)
Code 466.1	0 (0.0)	0 (0.0)	0 (0.0)	0 (0.0)	22 (0.9)	10 (0.4)	5 (0.4)
Code 466.11	302 (14.2)	220 (13.2)	8 (4.9)	426 (20.6)	632 (24.7)	697 (27.4)	268 (21.6)
Code 466.19	1821 (85.7)	1445 (86.6)	156 (95.1)	1641 (79.3)	1897 (74.2)	1832 (71.9)	962 (77.6)
Code 480.1	0 (0.0)	0 (0.0)	0 (0.0)	1 (0.0)	2 (0.1)	2 (0.1)	2 (0.2)
**Children 1–5 y**							
EDVs							
Code 07.96	1 (0.6)	0 (0.0)	0 (0.0)	2 (0.8)	7 (2.5)	17 (4.6)	7 (1.8)
Code 466.1	0 (0.0)	0 (0.0)	0 (0.0)	0 (0.0)	1 (0.4)	4 (1.1)	11 (2.8)
Code 466.11	14 (7.9)	19 (10.7)	2 (3.5)	51 (19.2)	39 (14.0)	75 (20.3)	102 (26.0)
Code 466.19	163 (91.6)	159 (89.3)	55 (96.5)	212 (80.0)	227 (81.7)	267 (72.2)	266 (67.7)
Code 480.1	0 (0.0)	0 (0.0)	0 (0.0)	0 (0.0)	4 (1.4)	7 (1.9)	7 (1.8)
Hospital admissions							
Code 07.96	0 (0.0)	0 (0.0)	0 (0.0)	2 (1.8)	1 (0.6)	3 (1.4)	2 (0.9)
Code 466.1	0 (0.0)	0 (0.0)	0 (0.0)	0 (0.0)	2 (1.1)	1 (0.5)	0 (0.0)
Code 466.11	7 (6.8)	17 (18.7)	2 (6.9)	20 (18.0)	36 (20.5)	69 (31.4)	68 (30.2)
Code 466.19	96 (93.2)	74 (81.3)	26 (89.7)	88 (79.3)	132 (75.0)	143 (65.0)	150 (66.7)
Code 480.1	0 (0.0)	0 (0.0)	1 (3.4)	1 (0.9)	5 (2.8)	4 (1.8)	5 (2.2)

*The following diagnosis codes used for the case definition of acute lower respiratory tract infection episodes, refers to infection caused by respiratory syncytial virus or RSV (07.96), acute bronchiolitis due to RSV (466.11), acute bronchiolitis due to other infectious organisms (466.19), and pneumonia due to RSV (480.1). EDV, emergency department visit; RSV, respiratory syncytial virus.

Appendix Table 6. Impact of universal RSV immunization on emergency department visits and hospitalizations for acute LRTI in children under 12 mo and those aged 1–5 y during the 2024–2025 winter season. The table reports acute LRTIs episodes, excluding diagnoses with ICD-9-CM code 07.96 (i.e., infection by respiratory syncytial virus).

	**Indicator activity, winter 2024–2025**		**Difference in indicator activity**	
Age cohort	Predicted (95% UI)	Observed	Absolute change	Percentage change (95% UI)
**Children <12 mo**				
EDVs	3,338 (3,059 to 3,617)	1,913	−1,425	−42.7 (−48.1 to −37.3)
Hospital admissions	2,313 (2,117 to 2,509)	1,237	−1,076	−46.5 (−51.9 to −41.1)
**Children 1–5 y**				
EDVs	278 (254 to 303)	386	108	38.8 (20.4 to 57.3)
Hospital admissions	158 (143 to 174)	223	65	41.1 (18.0 to 64.3)

*EDV, emergency department visit; LRTI, lower respiratory tract infection; UI, uncertainty interval

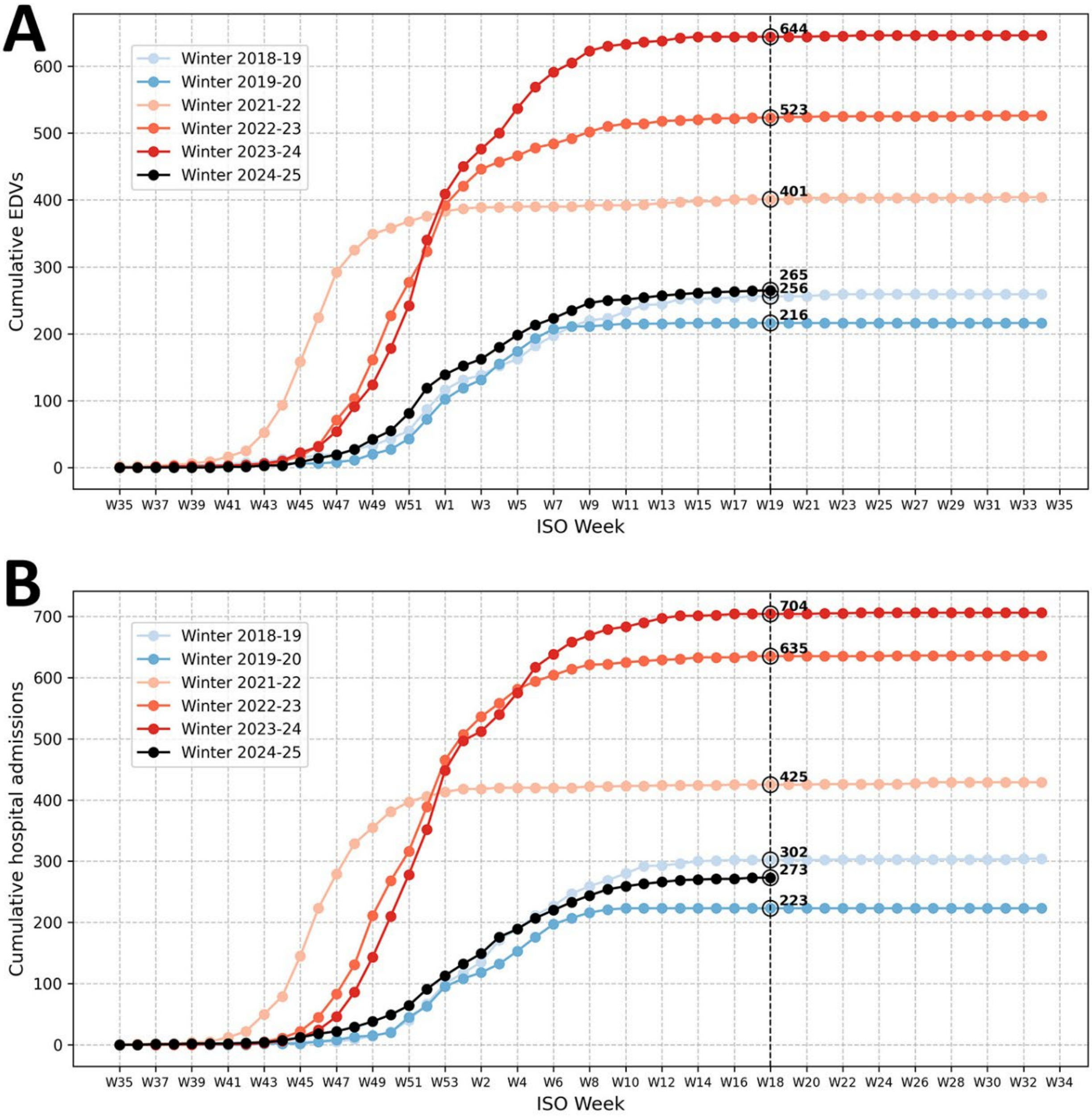

Appendix Figure 1. Comparison of cumulative emergency department visits and hospitalizations for RSV infection among children under 12 months across winter seasons. Cumulative weekly numbers of Emergency Department visits (**A**) and hospital admissions (**B**) for RSV infection among children aged below 12 months. To allow comparison between different epidemic years, this was defined as the period going from the 35th week of 1 year to the end of the 34th week of the following year, covering the entire winter epidemics and accounting for shifts in its onset and conclusion. Pre-COVID seasons are colored with different shades of blues, while post-COVID seasons with shades of reds. EDV = Emergency Department Visit.

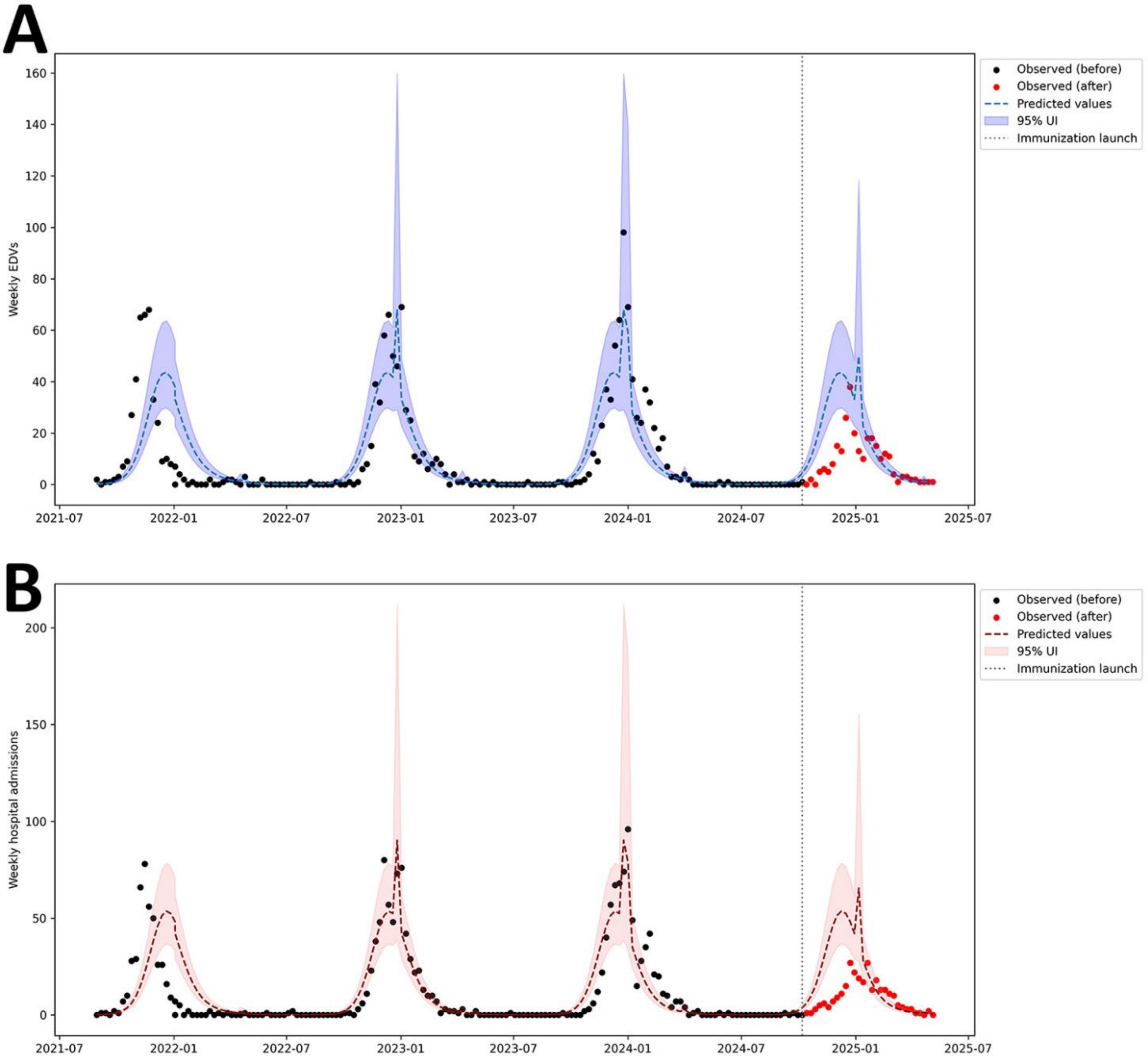

Appendix Figure 2. Interrupted time series analysis of Emergency Department visits and hospital admissions for respiratory syncytial virus infections among infants aged <12 months from ISO week 35–2021 to ISO week 19–2025. A negative binomial model with a Fourier parameter was trained with historic data before the launch of the universal RSV immunization program (before October 10, 2024) and used to compute predicted values and 95% uncertainty intervals (UI). This was displayed against the observed weekly counts of Emergency Department visits (**A**) and hospital admissions (**B**) for RSV infections.

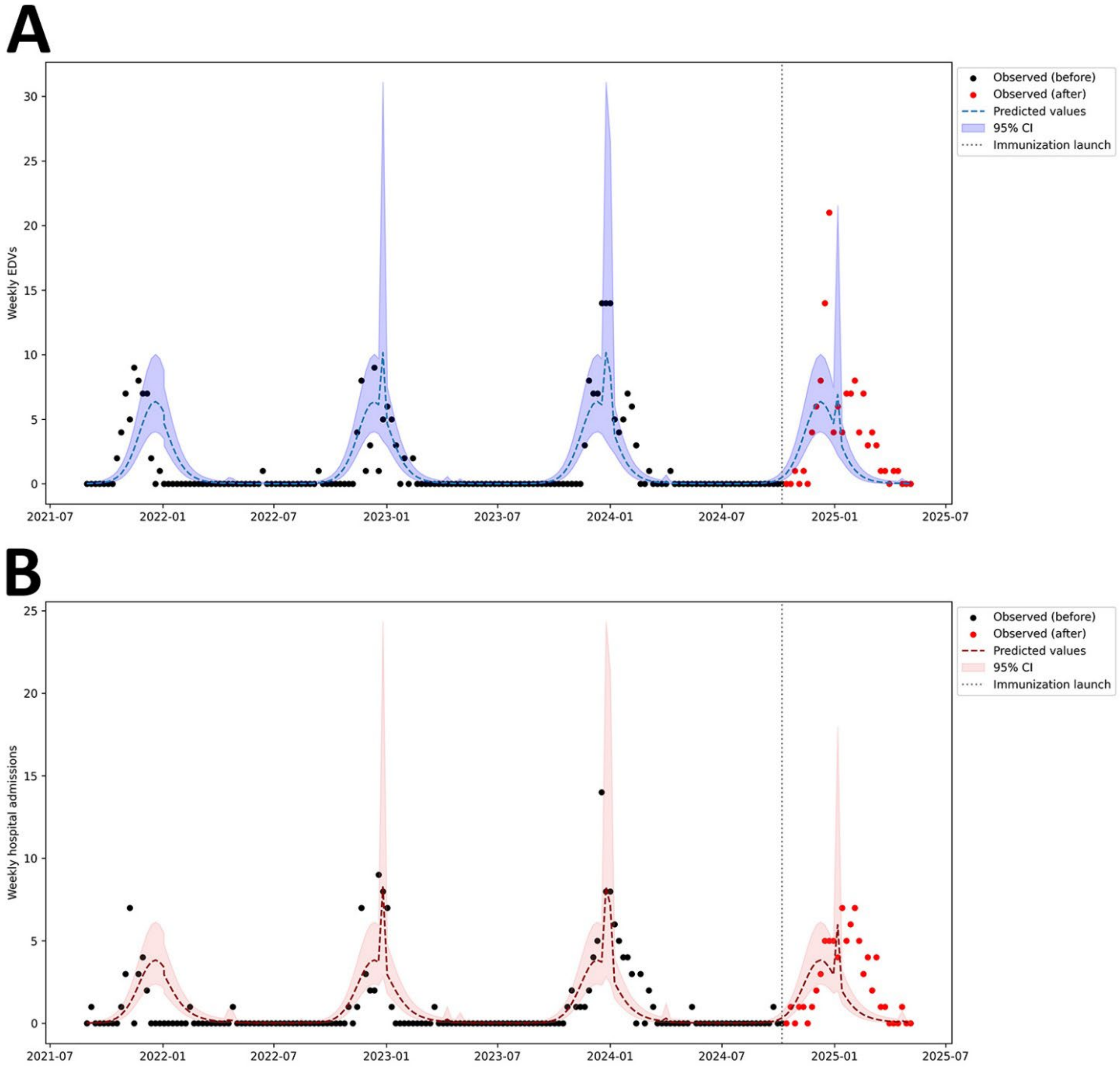

Appendix Figure 3. Interrupted time series analysis of Emergency Department visits and hospital admissions for respiratory syncytial virus infections among infants aged 1–5 years from ISO week 35–2021 to ISO week 19–2025. A negative binomial model with a Fourier parameter was trained with historic data before the launch of the universal RSV immunization program (before October 10, 2024) and used to compute predicted values and 95% uncertainty intervals (UI). This was displayed against the observed weekly counts of Emergency Department visits (**A**) and hospital admissions (**B**) for RSV infections.

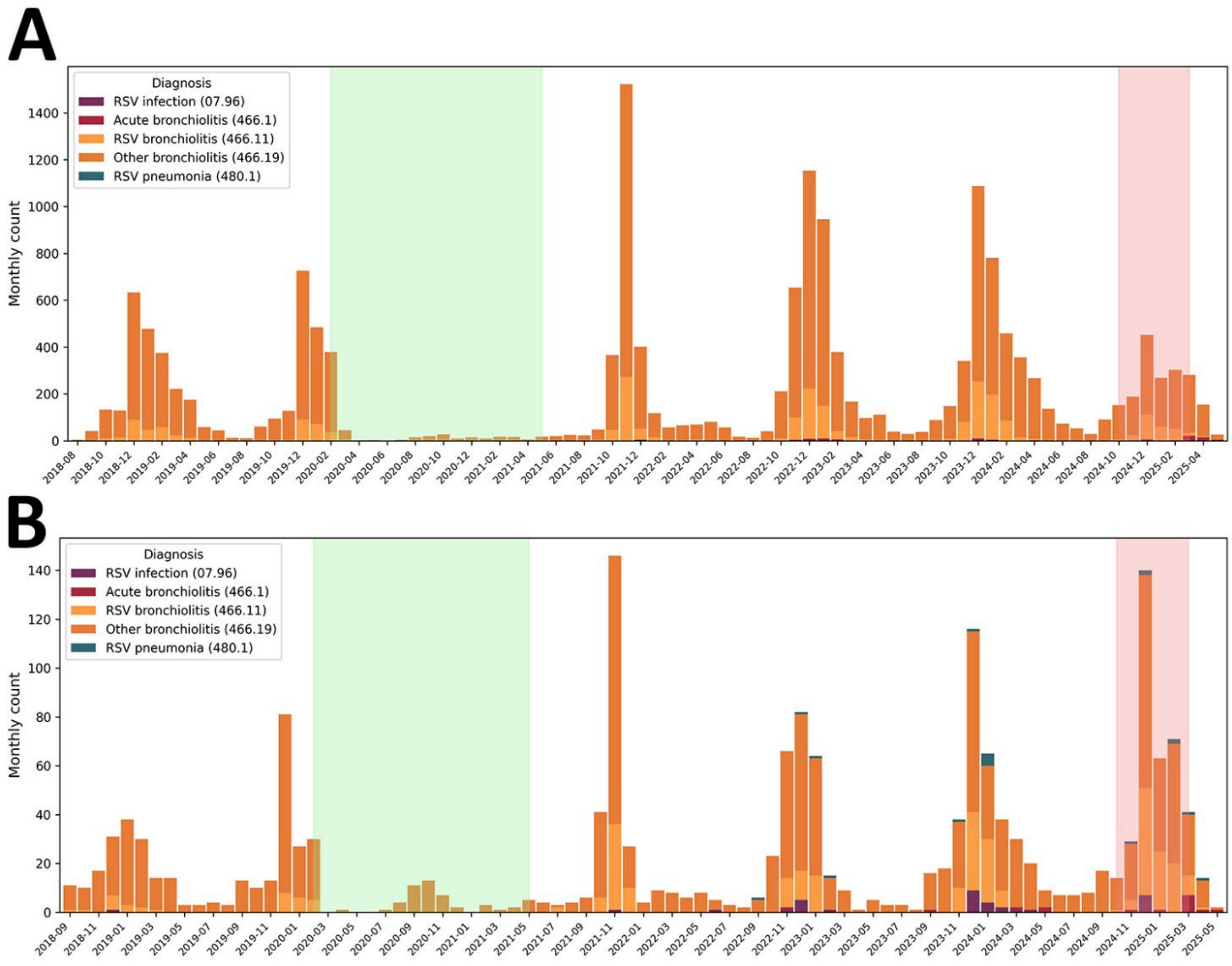

Appendix Figure 4. Monthly emergency department visits for acute lower respiratory tract infections among children under 12 months and between 1–5 years of age from ISO week 35–2018 to ISO week 19–2025. Monthly numbers of emergency department visits for lower respiratory tract infection among (**A**) children under 12 months of age and (**B**) children aged 1–5 years. The green-shaded area indicates the period during which public health and social measures were implemented in response to the initial waves of the SARS-CoV-2 in the region. The red-shaded area marks the prophylaxis period during which nirsevimab was administered.

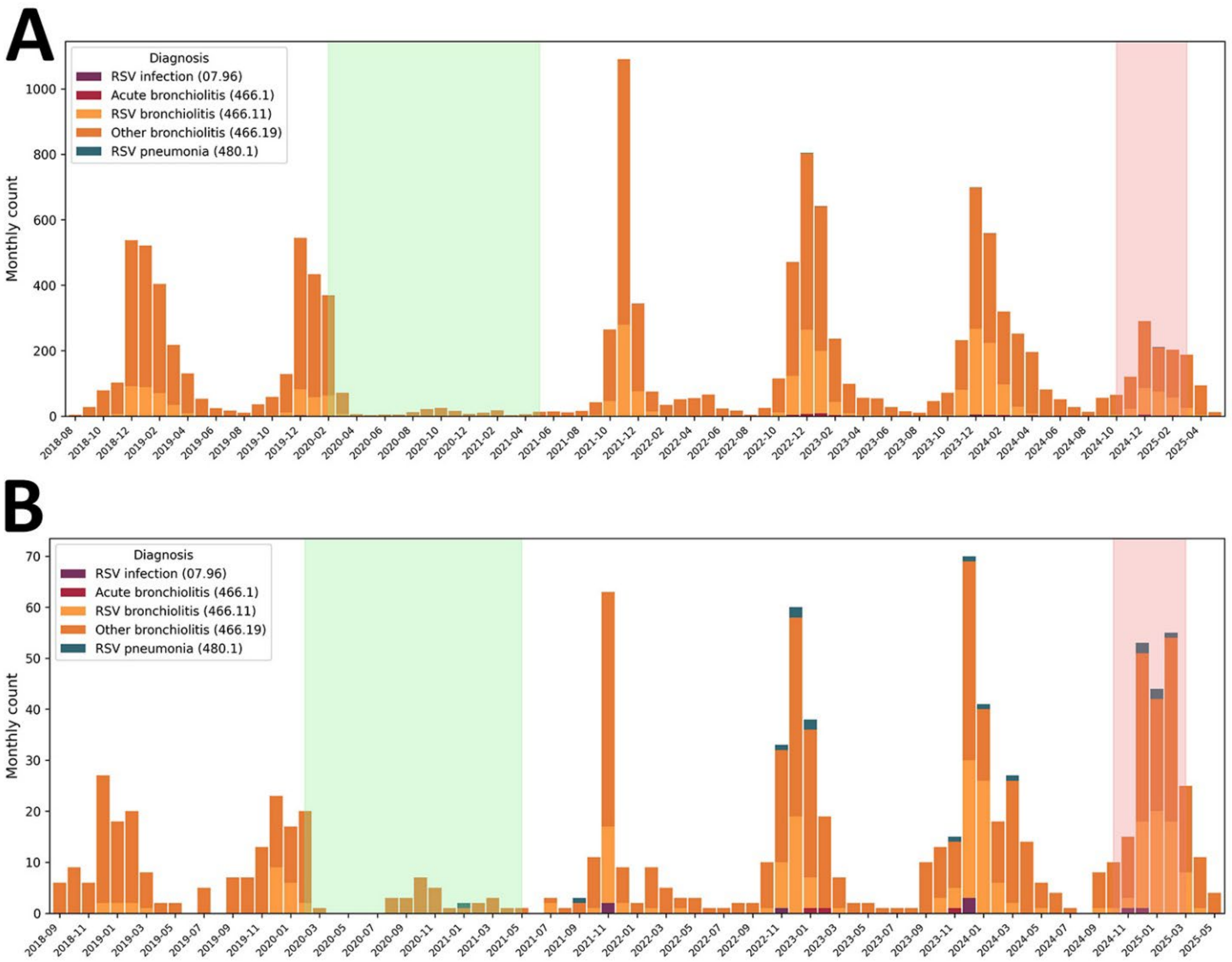

Appendix Figure 5. Monthly hospital admissions for acute lower respiratory tract infections among children under 12 months and between 1–5 years of age from ISO week 35–2018 to ISO week 19–2025. Monthly numbers of hospital admissions for lower respiratory tract infection among (**A**) children under 12 months of age and (**B**) children aged 1–5 years. The green-shaded area indicates the period during which public health and social measures were implemented in response to the initial waves of the SARS-CoV-2 in the region. The red-shaded area marks the prophylaxis period during which nirsevimab was administered.
